# The crosstalk between exosomes and ferroptosis: a review

**DOI:** 10.1038/s41420-024-01938-z

**Published:** 2024-04-09

**Authors:** Jiao Wu, Zhongyu Li, Yu Wu, Ning Cui

**Affiliations:** 1 Oncology Department of Xiyuan Hospital, China Academy of Chinese Medical Sciences, Beijing, China; 2 Department of Internal Medicine, Eye Hospital China Academy of Chinese Medical Sciences, Beijing, China

**Keywords:** Necroptosis, Oncogenesis

## Abstract

Exosomes are a subtype of extracellular vesicles composed of bioactive molecules, including nucleic acids, proteins, and lipids. Exosomes are generated by the fusion of intracellular multivesicular bodies (MVBs) with the cell membrane and subsequently released into the extracellular space to participate in intercellular communication and diverse biological processes within target cells. As a crucial mediator, exosomes have been implicated in regulating ferroptosis—an iron-dependent programmed cell death characterized by lipid peroxide accumulation induced by reactive oxygen species. The involvement of exosomes in iron, lipid, and amino acid metabolism contributes to their regulatory role in specific mechanisms underlying how exosomes modulate ferroptosis, which remains incompletely understood, and some related studies are still preliminary. Therefore, targeting the regulation of ferroptosis by exosomes holds promise for future clinical treatment strategies across various diseases. This review aims to provide insights into the pathophysiology and mechanisms governing the interaction between exosomes and ferroptosis and their implications in disease development and treatment to serve as a reference for further research.

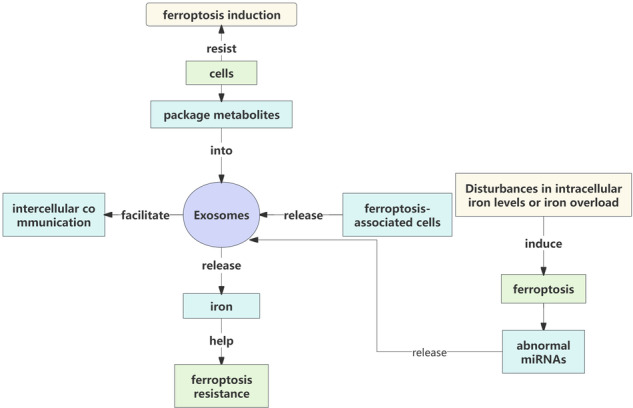

## Facts

- Exosome biogenesis is intricately regulated by a repertoire of ferroptosis-related proteins and protein complexes. There is an intersecting network of pathways governing both exosome release into the extracellular space and their degradation within lysosomes, which ultimately determine their distinct “fates”.
- The dynamic interplay between exosome biogenesis and ferroptosis is highly influenced by environmental cues, thus holding significant implications for normal physiological processes and pathological conditions.
- The involvement of exosomes in iron, lipid, and amino acid metabolism contributes to their regulatory role in specific mechanisms underlying how exosomes modulate ferroptosis, which remains incompletely understood.
- Although some related studies are still preliminary, targeting the regulation of iron death by exosomes holds promise for future clinical treatment strategies across various diseases. However, further validation through relevant clinical studies is warranted.
- This article comprehensively examines the pathophysiological mechanism underlying the intricate interplay between exosomes and ferroptosis over the past decade, elucidating its profound implications in disease development and treatment, thereby providing valuable insights for future investigations.

## Introduction

Extracellular vesicles (EVs) are membrane-encapsulated particles released into the extracellular environment by eukaryotic and prokaryotic cells [1]. The EVs can be categorized into exosomes, microvesicles, and apoptotic vesicles based on their molecular size and secretion mode [2]. Exosomes, crucial for intercellular communication, are bilayer lipid membrane particles of 30–100 nm diameter. They are widely present in various body fluids, including blood, urine, saliva, breast milk, amniotic fluid, ascites, cerebrospinal fluid, bile, and semen [3]. Exosomes can deliver maternal cell contents, including proteins, lipids, and nucleic acids (like DNA, mRNA, microRNA (miRNA), and other non-coding RNAs) to target cells for regulating their function [4, 5]. Exosomes, specifically internalized by target cells for intercellular crosstalk purposes, serve as biological messengers facilitating information communication [6, 7]. By transmitting proteins, metabolites, and nucleic acids that modulate recipient cell responses, exosomes can either promote or inhibit disease development [8, 9]. Associated with virtually all physiological and pathological cellular actions like stem cell maintenance [10], tissue repair [11], immune surveillance [12], tumor metastasis [13], pathogen infection [14], and others, exosomes possess intrinsic properties that enable them to regulate complex intracellular pathways, thereby enhancing their potential in disease treatment.

Ferroptosis is an emerging form of regulated cell death characterized by iron-dependent lipid peroxidation, which has garnered increasing attention from researchers [15]. Regarding morphology, molecular mechanisms, and immunological characteristics, ferroptosis is distinguished from other forms of cell death, like apoptosis, programmed necrosis, and autophagy [16]. Furthermore, ferroptosis plays a significant role in the pathogenesis of various diseases and tumor resistance [17, 18]. While previous studies have primarily focused on exosomes derived from activated or apoptotic cells, with limited consideration for necrotic cells [19], recent research has focused on the intricate molecular regulatory mechanism between exosomes and ferroptosis [19, 20], thereby opening up new avenues for disease treatment. Consequently, exploring theosomes and ferroptosis represents a pivotal breakthrough in disease management with profound implications for human health, which may become a new field for disease treatment.

## Exosomes

### Discovery and mechanism of exosomes

In 1983, Johnstone et al. first observed the release of membrane vesicles from mature reticulocytes in sheep, which were initially believed to be involved in expelling excess transferrin (Tf) receptors and subsequently named “exosomes” [21]. Exosomes are nanoscale extracellular vesicles derived from the endocytic pathway [22]. Endocytosis is a cellular process whereby cytoplasm, macromolecules, membranes, and receptors are internalized through plasma membrane invagination, resulting in intracellular vesicle formation upon membrane breakage. Primary endocytic vesicles merge with early endosomes to initiate the sorting of cargo molecules [23]. Early endosomes mature into late endosomes and eventually fuse with lysosomes. During maturation, some endosomes generate intermediate organelles characterized by luminal vesicles within tubular structures, known as multivesicular bodies (MVBs). The MVBs can either fuse with lysosomes/autophagosomes for degradation or fuse with the plasma membrane to release intraluminal vesicles, including exosomes, into the extracellular space [1, 24]. Exosome generation is a continuous process influenced by factors, including free radical stress, ultravoilet radiation, membrane cholesterol levels, intracellular calcium levels, and others [25, 26]. Exosomes have been detected in vivo within living cells and in vitro cultured cells, including adipocytes, tumor cells, and mesenchymal stem cells (MSCs) [27]. Furthermore, research has shown that exosomes can be isolated from various body fluids, including human blood, urine, amniotic fluid, and ascites [28, 29], indicating that exosome secretion represents a universal cellular function rather than being solely a metabolic product associated with normal cellular physiology [30, 31].

Morphologically, exosomes exhibit a lipid bilayer structure with a diameter ranging from 40–100 nm [32]. They also harbor an abundance of membrane-associated proteins, including four-transmembrane proteins (cluster of differentiation 63 (CD63), CD81, and CD9) and heat shock proteins (Hsp60, Hsp70, and Hsp90). Furthermore, exosomes express Alix, the tumor susceptibility gene 101, and the lattice protein [33]. Exosomes encompass various biologically active substances, including cytokines, lipids, mRNA molecules, and non-coding RNA species [27]. Exosomes are enclosed within a dual membrane envelope that safeguards their cargo while facilitating long-distance intercellular transport through tissues [34]. Exosomes possess dynamic contents that change depending on cell type and state.

### Mechanisms of exosome regulation

**Fig. 1:**
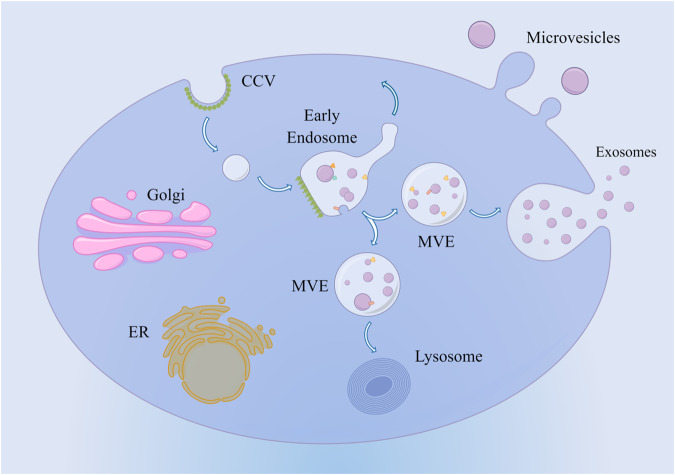
Molecular mechanism of exosomes.

Exosomes exhibit wide distribution and functional diversity, closely associated with their respective derived cells [35]. Their biological characteristics are influenced by various factors, including contents, tissue microenvironment, and receptor cells, resulting in diverse effects. Exosomes possess an enriched surface composition of cholesterol, diglycerides, sphingolipids, phospholipids, and other lipids that maintain their morphology and serve as signaling molecules in numerous biological processes [36]. Exosomes can enter target cells through diffusion, endocytosis, or receptor-mediated mechanisms and transport specific proteins, lipids, mRNAs, and miRNAs along with other biologically active substances to perform diverse biological functions [37]. Specifically, genetic information is also transferred from parent to recipient cells *via* exosomes: the lipid bilayer membrane of exosomes protects mRNA and miRNA from degradation by ribonucleases. Consequently, they remain stable in blood and body fluids. Upon engulfment by receptor cells, exosomes release their contents, including mRNAs and miRNAs, into the cytoplasm, thus facilitating information exchange and modulation of gene expression [38–40]. Moreover, exosome-mediated intercellular communication occurs through the exosome membrane ligand binding to target cell membrane receptors; for instance, Wnt-carrying exosomes induce Wnt signaling activity in target cells [41]. In addition, intracellular substances released by exosomes affect target cell receptors, leading to information transfer completion and generation of biological effects; for instance, active components within exosomes bind to endothelial cell surface receptors, promoting angiogenesis [42]. It can be concluded that the functionality mechanism exhibited by exosomes is determined based on maternal and recipient cell types and their physiopathological state. The mechanism is illustrated in Fig. 1.

## Ferroptosis

### Ferroptosis: mechanisms and links with diseases

Ferroptosis, first described in 2012 as a form of cell death induced by the small molecule erastin [15], is characterized by cystine input inhibition, resulting in glutathione (GSH) depletion and inactivation of the phospholipid peroxidase glutathione peroxidase 4 (GPX4) [43]. It exhibits distinct morphological, biochemical, and genetic features compared to other forms of cell death. Morphologically, ferroptotic cells display necrotic-like changes like loss of plasma membrane integrity, cytoplasmic and organelle swelling, and chromatin condensation [44]. Ultrastructurally, it is characterized by alterations in mitochondrial structure, including condensed mitochondria with reduced or absent cristae and increased membrane density along with the outer mitochondrial membrane rupture. However, it does not exhibit reduced nuclear volume or staining cohesion, which are commonly used markers for detecting ferroptosis occurrence [15, 45]. Biochemically, key features include decreased synthesis or depletion of the antioxidant GSH and diminished activity of the lipid repair enzyme GPX4 on membranes, leading to reactions resulting in iron ions-mediated accumulation of toxic lipid peroxides. Genetically, ferroptosis involves a signaling cascade regulated by multiple metabolic pathways, including oxidative stress response mechanisms and iron and lipid metabolism [46–48].

### Mechanism of ferroptosis

#### Lipid peroxidation pathway

Lipid peroxidation refers to the oxidation, fragmentation, and shortening of polyunsaturated fatty acids (PUFA) or allyl group-rich phosphatidylethanolamine on biological lipid membranes under the influence of oxygen free radicals. This process generates cytotoxic substances, including lipid free radicals, lipid hydroperoxides, and reactive aldehydes (malondialdehyde and 4-hydroxynonenal), which ultimately lead to cell disintegration and death by disrupting the cell’s lipid bilayer, protein, and DNA structure. The Acyl-coa synthetase long-chain family member 4 (ACSL4) and lysophosphatidylcholine acyltransferase 3 (LPCAT3) are two crucial enzymes involved in the synthesis of PUFA-PLs within the endoplasmic reticulum. They activate PUFAs, modulate their transmembrane properties, and enhance cellular sensitivity to ferroptosis, lipoxygenase (LOX) plays the most important role in the process of lipid peroxidation [49, 50], with 15-lipoxygenase-1 (15LO1) being one of its key enzymes. During arachidonic acid (AA) metabolism, oxygen molecules are inserted into PUFA to form hydroperoxide eicosapentaenoic acid, subsequently reduced to 15-hydroxyeicosatetraenoic acid. Conversely, acyl-CoA synthetase long-chain family member 3 or stearoyl-CoA desaturase (SCD/SCD1) inhibits ferroptosis in cancer cells [51]. Lipid metabolism disorders are closely associated with ferroptosis since lipid oxides and reactive oxygen species (ROS) are prerequisites for this process.

#### Iron metabolic pathways

Iron homeostasis is a complex process that relies on the regulation of various iron metabolism proteins. An imbalance in iron homeostasis can lead to iron overload and ferroptosis [52]. Cellular iron homeostasis is associated with the interplay between cellular iron uptake, storage, and export. The extracellular trivalent iron enters the intracellular compartment through the Tf and Tf receptor 1 (TFR1) complex [53], where it is reduced to a dynamic pool of iron by metal reductase 3 and transported into the cytoplasm *via* divalent metal transporter (DMT1) [54]. In cases of intracellular iron overload, excess Fe^2+^ catalyzes lipid peroxide generation and hydroxyl radical formation through the Fenton reaction, ultimately leading to ferroptotic cell death [55]. Simultaneously, free radicals generated during this process also inflict damage upon intracellular proteins, nucleic acids, and lipids, further promoting ferroptosis occurrence [56]. In addition, nuclear receptor coactivator 4, acting as a ferritin mediator *via* autophagic degradation pathway, increases intracellular free Fe^3+^ levels, which subsequently reduces to Fe^2+^ by STEAP3, leading to intracellular iron overload while enhancing cell sensitivity towards ferroptosis [52, 57]. Iron can also flux into unstable pools within organelles for potential. Excessively accumulated iron may be exported out of cells *via* ferroportin 1 (FPN1), which serves as the sole known protein controlling mammalian cellular iron export crucial for overall systemic balance [58]. Perturbations in both increased uptake and decreased export contribute to disrupted equilibrium in cellular iron metabolism, resulting in detrimental consequences. Although the precise mechanisms underlying iron’s involvement in ferroptosis remain elusive, its indisputable role as a critical mediator of ROS production underscores its significance.

#### Signaling pathways of cystine/glutamate transport receptor (System x_c_^–^) and GSH-GPX4 antioxidant systems inducing ferroptosis

The System x_c_^–^ is a membrane Na^+^-dependent amino acid antiporter, comprising heterodimers S from solute carrier family 7 member 11 and solute carrier family 3 member 2, respectively [59]. This system facilitates cysteine transportation, a raw material for GSH synthesis, into cells by exchanging glutamic acid and cystine at a 1:1 ratio while reducing cystine to cysteine. In addition, it participates in GSH synthesis [15]. The GPX4 is crucial for in vivo lipid peroxide hydrolysis by directly reducing hydrogen peroxide phospholipids to hydroxyphospholipids, maintaining intracellular free radical content balance, and regulating ferroptosis [60]. The cofactor glutathione, dependent on GPX4, reduces toxic lipid peroxides to non-toxic lipid alcohols that repair damaged biofilms and prevent ferroptosis [61]. Pharmacological inhibition, genetic inactivation, or low expression of GPX4 can decrease cellular antioxidant capacity while increasing lipid ROS levels, ultimately leading to cell ferroptosis [62].

#### Other ways of inducing ferroptosis

**Fig. 2:**
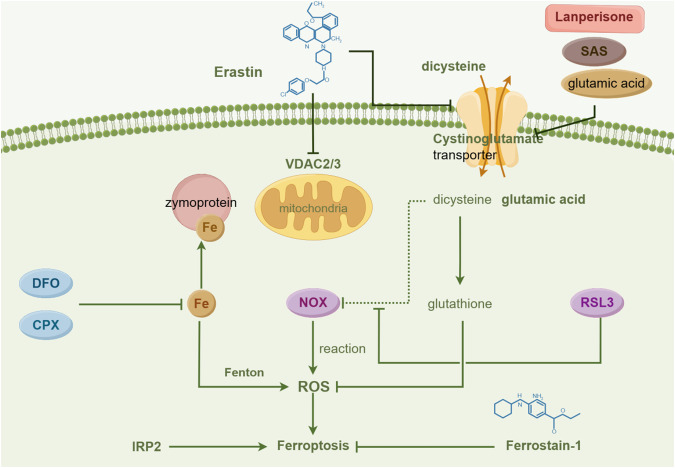
The molecular mechanisms underlying ferroptosis.

The TP53 variations (mutations or polymorphisms) can modulate p53’s ability to induce apoptosis and ferroptosis, while TP53 mediates SLC7A11 transcriptional repression to promote ferroptosis in cancer cells [63]. Hypoxia can trigger tumor formation and treatment resistance, with oxygen-inducible transcription factors playing a dual role in regulating ferroptosis and maintaining lipid homeostasis [64]. The CD44-mediated enhancement of iron endocytosis boosts iron-dependent demethylase activity, thereby promoting epithelial–mesenchymal transition (EMT) signaling-related gene expression and sensitizing cancer cells to ferroptosis [65]. The mechanism of ferroptosis is illustrated in Fig. 2.

## Crosstalk between ferroptosis and exosomes

Cellular iron transport and detoxification primarily rely on the function of ferritin, a 450 kDa protein complex comprising 24 light and heavy chain subunits [66]. As a ferrous oxidase, ferritin heavy chain (FTH) generates trivalent iron within the core of ferritin and has long been recognized as an intracellular iron storage protein [67]. Recent studies have unveiled two potential mechanisms for ferritin secretion: exosome-mediated and autophagy-mediated pathways [68, 69]. The Tf and lactoferrin, the iron carrier molecules, are found in exosomes and can be internalized by mammalian cells through the MVB-exosome pathway [69, 70]. Ferritin has also been identified in human urinary exosomes through large-scale proteomic analyses [71, 72], where it binds to iron *via* exosomal secretion. This ability of exosomes to transfer iron carrier molecules between cells may be crucial for intercellular communication and maintaining physiological iron homeostasis. Furthermore, exosomal iron release contributes to ferroptosis resistance [73], like Prominin-2, a lipid kinetic regulatory protein that enhances MVB formation and facilitates the transportation of ferritin-containing exosomes to extrude cellular iron, thereby preventing ferroptosis [20]. These findings indicate that exosomes can regulate ferroptosis while playing a significant role in conferring resistance against it [74].

Contrarily, ferroptosis-associated cells can release exosomes that facilitate intercellular communication. For instance, in an infarction model, ferroptosis-associated cardiomyocyte-derived exosomes were observed to activate the Wnt/β-catenin signaling pathway in cardiac macrophages, thereby promoting the pathological progression of myocardial infarction (MI) towards the M1 macrophage polarization phenotype [75]. Disturbances in intracellular iron levels or iron overload can induce cellular ferroptosis and result in the release of granulocyte exosomes containing abnormal miRNAs, which subsequently impact disease progression [76]. In endometriosis patients, iron overload has been demonstrated to cause aberrant miRNA components within oocyte maturation by affecting cell cycle regulation, oocyte meiosis, and ferroptotic signaling pathways [77]. Interestingly, due to their resistance to ferroptosis induction, cells also package metabolites into exosomes and expel them outside as a defense mechanism against external stimuli [20]. Overall, a synergistic interaction between exosomes and cellular ferroptosis exists that holds significant implications for understanding regulatory mechanisms when either or both are dysregulated.

## Role of exosomes and ferroptosis in disease occurrence

### Cancer

Cancer is a prevalent and lethal disease worldwide; however, current therapeutic modalities, including surgery, chemotherapy, radiotherapy, targeted drugs, and immunotherapy, have limited efficacy in treating cancer. Therefore, exploring alternative strategies to induce cancer-specific cell death and overcome anti-tumor drug resistance is imperative. Exosomes are crucial for promoting chemoresistance in tumor cells; thus, comprehending the underlying mechanisms of this resistance can significantly enhance treatment outcomes and prognosis. Notably, exosomes derived from cancer-associated fibroblasts (CAFs) contribute to tumor metastasis and augment chemoresistance in cancer cells [78, 79]. Zhang et al. demonstrated that chemotherapeutic drug toxicity stimulates CAFs to secrete exosomal miR-522, which inhibits ferroptosis in gastric cancer cells by targeting ALOX15 and impeding lipid-ROS accumulation [80]. This intercellular pathway involving exosomal miR-522 and ferroptosis unveils a novel mechanism of acquired chemotherapy resistance in gastric cancer. In addition, Zhang et al. discovered that exosome lncFERO derived from gastric cancer cells enhances the stearoyl-coenzyme A desaturase-1 protein expression while recruiting heterogeneous nuclear ribonucleoprotein A1 (hnRNPA1), leading to dysregulated PUFA levels and suppressed ferroptosis activity, thereby reducing chemosensitivity of cancer stem cells (CSCs) [81]. Consequently, targeting the exosome-mediated crosstalk between ferroptosis pathways holds promise as an effective approach for clinically managing solid tumors and preventing chemotherapy resistance.

Exosomes and ferroptosis both play crucial roles in lung cancer pathogenesis. Zhang et al. demonstrated that exosomal RNA-protein interactions can regulate ferroptosis in lung adenocarcinoma, potentially through the interaction between exosomal circRNA_101093 (cir93) and fatty acid binding protein 3 (FABP3). The FABP3 facilitates the AA transport and its reaction with taurine, resulting in reduced overall AA levels and lipid peroxidation and desensitization of lung adenocarcinoma cells to ferroptosis [75]. Therefore, combination therapy targeting exosome biosynthesis and function may offer improved treatment strategies for lung adeninoma. Moreover, miRNA transfer *via* exosomes influences recipient cell proliferation and chemoresistance. Song et al. identified elevated levels of microRNA-4443 (miR-4443) within non-small cell lung cancer (NSCLC) tumor-derived exosomes as a negative regulator of ETLL3-induced modification of human fibroblast-specific protein 1 m6A methylation. This regulatory mechanism mediates ferroptosis induction while promoting tumor growth [82]. With an enhanced comprehension of chemoresistance and the validation of therapeutic potential in targeting ferroptosis, the role of ferroptosis in tumor radiosensitivity has emerged as a captivating subject [83]. Zhang et al. found that exosomes derived from hypoxic NSCLC cells can directly transmit radioresistance to surrounding oxygenated NSCLC cells in an exosomal ANGPTL4-GPX4-dependent manner, thereby inducing radioresistance in the receptor [84]. These findings collectively demonstrate the targeted role of exosomal RNA-induced ferroptosis in impeding tumor cell proliferation and metastasis and overcoming chemotherapy resistance in malignant tumors.

Exosomes also exert anti-tumor effects by inducing ferroptosis in cancer cells, mediating cellular immune responses, and regulating the tumor microenvironment. Kirsten rat sarcoma viral oncogene (KRAS) plays a pivotal role in cancer-macrophage communication, with KRASG12D protein being released from autophagy-dependent ferroptotic cancer cells through an oxidative stress response. The uptake of exosomal KRASG12D by macrophages activates signal transducer and activator of transcription 3 (STAT3)-dependent fatty acid oxidation, leading to the acquisition of an M2-like pro-tumor phenotype, promoting M2 macrophage polarization, and stimulating macrophage-induced pancreatic tumor growth [19]. Hu et al. discovered that exosomes derived from HBV-positive liver cancer cells containing miR-142-3p induce ferroptosis in M1 macrophages SLC3A2, thereby facilitating liver cancer progression [85]. Li et al. discovered that the hepatocellular carcinoma (HCC)-targeted exosome SP94-Lamp2b-RRM facilitates the small interfering RNA delivery to enhance sorafenib-induced ferroptosis by silencing GPX4 and DHODH expression. This mechanism increases HCC sensitivity to sorafenib [86]. This novel finding unveils a promising avenue for overcoming sorafenib resistance in HCC through ferroptosis modulation. Zhang et al. revealed that adipose-derived exosomes microsomal triglyceride transfer protein inhibit zinc finger E-box binding homeobox 1 expression while upregulating GPX4 and xCT, resulting in reduced lipid ROS production and decreased PUFA levels, which effectively suppress iron-mediated cell death and diminish chemotherapy sensitivity in colorectal cancer [87]. Chen et al. identified high levels of macrophage migration inhibitory factor within exosomes secreted by nasopharyngeal carcinoma cells, which macrophages can internalize to inhibit iron-mediated cell death and consequently promote metastasis of nasopharyngeal carcinoma [88]. These studies collectively demonstrate the ability of exosomes to induce ferroptosis in cancer cells by impeding tumor proliferation and metastasis, thus providing valuable insights and experimental foundations for combating tumor chemotherapy resistance.

### Liver and kidney diseases

Currently, the shortage of donor livers poses a significant constraint on clinical liver transplantation, and researchers are actively seeking more effective approaches to address this issue [89]. The MSCs are considered an ideal cell source for tissue regeneration and have shown potential in promoting liver regeneration and repairing liver injury [90, 91]. Wu et al. discovered that exosomes derived from hemeified bone marrow MSCs (HO-1/BMMSCs) effectively suppressed hepatocyte ferroptosis by delivering miR-124-3p, thereby significantly reducing the iron homeostasis factor prostaglandin VI transmembrane epithelial antigen 3 [92]. This approach also mitigated post-transplant inflammatory response and provided a novel direction for addressing ischemia-reperfusion injury (IRI) in liver transplantation [92]. In addition, Li et al. demonstrated that HO-1/BMMSC-derived exosomes containing miR-29a-3p alleviated hepatic IRI by negatively regulating IREB2 protein expression, reducing intracellular Fe^2+^ levels, promoting lipid ROS production, and inhibiting ferroptosis [5].

Hepatic fibrosis is characterized by abnormal connective tissue proliferation in the liver, resulting from various pathogenic factors. The key factor involved is the activation of hepatic stellate cells (HSCs) [93]. Studies have demonstrated that HSCs store significant amounts of ions [94], creating a conducive environment for ferroptosis in these cells. Hepatic crosstalk between exosomes and MSCs has been shown to improve liver fibrosis [95, 96]. Tan et al. discovered that MSCs-derived exosomes (MSC-Exo) deliver BECN1 protein to activated HSCs, thereby promoting xCT/GPX4-mediated ferroptosis in HSCs [97]. This novel finding highlights the role of MSC-Exo in ferroptosis during liver fibrosis. Furthermore, MSC-released exosomes contain specific types of RNA, lipids, and proteins essential for physiological homeostasis, cell proliferation, and tissue regeneration [98]. Lin et al. found that MSC-Exo enhances SLC7A11 protein stability, activating System x_c_^-^ and protecting against hepatocyte ferroptosis [99]. Collectively, these studies demonstrate how exosomes can influence hepatic iron death while ameliorating IRI and fatty liver-induced liver fibrosis, thereby offering promising therapeutic potential for clinical applications. Current basic research suggests that targeting the interaction between MSC-Exo and ferroptosis may help mitigate liver injury. In addition, baicalein-pretreated MSCs exhibit a protective effect on hepatic function by activating the Kelch-like epichlorohydrin-related protein 1-nuclear factor E2-related factor 2 pathway *via* the P62 signaling cascade, which inhibits ROS production and lipid peroxide-induced ferroptosis [100]. These findings highlight the potential development of baicalin as a drug.

Furthermore, studies have demonstrated the potential of human urine-derived stem cells (USCs) in ameliorating renal function and pathomorphology in IRI-induced acute kidney injury (AKI) [101, 102]. Sun et al. discovered that human USC-derived exosomes (USC-Exo) carry long non-coding RNA (lncRNA) TUG1, which regulates Acyl-CoA synthetase long-chain family member 4 (ACSL4) -mediated ferroptosis through interaction with SRSF1, thereby protecting against renal IRI-induced AKI [6]. Notably, USC-Exo enriched with lncRNA TUG1 holds promise as a potential therapeutic approach for IRI-induced AKI.

### Cardiovascular disorders

The heart can be affected by excessive iron accumulation, ROS production, and pathological transformation of membrane lipids, all of which are crucial factors in ferroptosis development. Exosomal regulation of ferroptosis is involved in the pathophysiological processes of cardiovascular diseases and plays a significant role in myocardial damage. Studies have demonstrated that IRI exacerbates functional and structural damage during tissue and organ reperfusion after ischemia [103, 104]. Zhang et al. elucidated that exosomes derived from bone marrow MSCs effectively enhance cardiomyocyte proliferation while attenuating ischemia-reperfusion-induced cardiac injury by inhibiting cardiomyocyte ferroptosis *via* modulating Pum2/PRDX6 axis [105]. Consequently, this approach reduces myocardial IRI and ameliorates ischemic conditions within myocardial tissue. Furthermore, exosomes derived from adipose tissue macrophages are implicated in obesity-induced ferroptosis and cardiac injury [106]. For instance, Zhao et al. discovered that miR-140-5p carried by adipose tissue macrophage-derived exosomes could participate in obesity-induced cardiac injury by targeting downstream SLC7A11 to suppress GSH synthesis and induce ferroptosis [106]. This presents a novel therapeutic strategy for targeting obese adipose tissue macrophage-derived exosomes in obesity-related myocardial injuries. Song et al. demonstrated that exosomes derived from human umbilical cord blood mesenchymal stromal cells exhibited a significant tendency to inhibit the target gene transporter protein DMT1 expression through miR-23a-3p, thereby attenuating intracellular lipid oxidation and ultimately suppressing iron-induced cell death in cardiomyocytes, leading to improved cardiac function and histological injury [107]. In addition, Sun et al. identified that exosome-mediated communication between ferroptotic cardiomyocytes and macrophages activated the Wnt/β-catenin pathway, inducing M1 macrophage polarization and contributing to MI pathological progression [108]. This finding may offer a novel therapeutic target for heart attack treatment. Furthermore, interventions aimed at protecting peri-infarct cardiomyocytes are closely associated with cardiac remodeling during MI [109]. Man et al. discovered that pericardial adipose tissue-secreted exosomes loaded with adipokines interacted with iron regulatory protein 2 to safeguard cardiomyocytes against ferroptosis while maintaining iron homeostasis [110].

Chemotherapy drugs can induce cardiotoxicity mediated by ROS or oxidative stress. Exosomes serve as markers for doxorubicin-induced cardiotoxicity and can effectively function as drug delivery vehicles through specific proteins and genetic material (miRNA and lncRNA). They reduce ROS levels, inhibit cellular ferroptosis, and alleviate cardiotoxicity [111]. This study demonstrates the novel potential of exosome-based carriers in reducing chemotherapy-induced cardiotoxicity by acting as cellular iron death inhibitors. In addition, pacing cardiac fibroblasts secrete exo-miR-23a-3p to suppress SLC7A11 mRNA transcription, deplete the X_c_^−^ transport system, and increase ferrous ion concentrations and lipid peroxidation, thereby promoting ferroptosis in cardiomyocytes [112]. Intervening with exosomal miRNAs to mitigate cardiomyocyte loss and oxidative stress damage may prevent persistent atrial fibrillation development. The interplay between exosomes and ferroptosis is implicated in myocardial tissue damage, cardiotoxicity, and atrial fibrillation pathogenesis. These findings provide a new perspective on potential interventions for cardiovascular diseases.

### Neurological disorders

Exosomes are implicated in the pathogenesis of neurological disorders by regulating ferroptosis. Numerous studies have demonstrated the crucial role of exosomes in managing neurodegenerative diseases, nerve injuries, and other neurological disorders [113]. In neurodegenerative diseases, neurofilament light chain, a biomarker for axonal injury, can participate in non-classical neuronal ferroptosis by releasing FTH-containing exosomes from oligodendrocytes into the extracellular matrix, thereby influencing neuronal function [114]. Liu et al. discovered that MSC-Exo can ameliorate cognitive impairment by inhibiting hippocampal ferroptosis *via* activating the SIRT1/Nrf2/HO-1 pathway during delayed neurocognitive recovery [115]. These findings substantiate the potential therapeutic value of exosomes and offer novel insights into drug development for enhancing cognitive impairment.

Effective treatments for intracerebral hemorrhage (ICH) remain limited. Li et al. discovered that exosomal miR-137 overexpression in endothelial progenitor cells enhances neuroprotection against oxyhemoglobin (OxyHb)-induced mitochondrial dysfunction and upregulates OxyHb-induced miR-137 levels in endothelial progenitor cells, thereby inhibiting OxyHb-induced iron death and reducing the incidence of cerebral hemorrhage [116]. Furthermore, Yi et al. demonstrated that overexpression of adipose stem cell (ADSC)-derived exosome miR-19b-3p can directly target iron regulatory protein, leading to reduced ferroptosis-induced intracerebral hemorrhage and neurological damage [117]. These studies suggest that exosomes may mitigate cerebral hemorrhage by inhibiting ferroptosis, which could potentially identify novel therapeutic targets for hemorrhagic strokes.

Septic-associated encephalopathy is a cerebral clinical lesion associated with severe sepsis, an incompletely understood pathological condition. Wei et al. discovered that sepsis can induce upregulated expression of plasma exosome-derived nuclear enriched transcript 1, which competes with miR-9-5p in a competing endogenous RNA network, thereby enhancing the expression of Tf receptor and glutamate oxaloacetate transaminase 1 axis. Consequently, this decreases the GSH and GPX4 levels, ultimately promoting ferroptosis in brain microvascular endothelial cells and exacerbating sepsis [118]. This study highlights the regulatory role of exosomes in ferroptosis and suggests their potential as a novel therapeutic intervention for septic encephalopathy.

### Other diseases

Exosomes and iron-mediated cell death are pivotal in various diseases. Wang et al. demonstrated that alveolar macrophage-derived exosomes transfer tRF-22-8BWS7K092 to lung epithelial cells, activating the Hippo signaling pathway by binding to Wnt5B and promoting ferroptosis, thereby contributing to acute lung injury pathogenesis [119]. Endometrial regenerative cells (ERCs) are a novel source of adult stem cells with therapeutic effects in experimental colitis [120]. Zhu et al. found that ERC-derived exosomes (ERC-Exo) increase GSH and GPX4 expression in the colon of colitis while reducing iron, MDA, and ACSL4 expression to inhibit intestinal ferroptosis and alleviate ulcerative colitis (UC) [121], providing new insights into future UC treatment. Endometriosis is a significant risk factor for infertility [122]. Ni et al. discovered that iron-overloaded ovarian follicular fluid induces granulosa cell ferroptosis, releasing granulosa cell exosomes containing abnormal miRNAs that impair oocyte maturation in endometriosis [77].

## Prospects

Exosome biogenesis and ferroptosis play pivotal roles in maintaining cellular homeostasis and mitigating cellular stress, with mounting evidence suggesting that these cellular responses are achieved through intricate crosstalk between ferroptosis and exosomes. At the molecular level, proteins and protein complexes associated with ferroptosis contribute to exosome biogenesis. At the organelle level, the pathways of exosomes and ferroptosis intersect, determining their diverse “fates,” including extracellular release or lysosomal degradation. The dynamic and context-dependent interplay between exosome biogenesis and ferroptosis holds significant implications for both normal physiology and pathology. Therefore, gaining a comprehensive understanding of the mechanisms underlying their regulation is crucial for advancing clinical interventions, warranting future validation through relevant clinical studies.

## Data Availability

The raw data supporting the conclusion of this article will be made available by the authors, without undue reservation.
